# Impact of vitamin D level on glycemic control in diabetes mellitus type 2 in Duhok

**DOI:** 10.1016/j.amsu.2021.102208

**Published:** 2021-03-05

**Authors:** Yosuf Abdullah Salih, Mohammed T. Rasool, Idris Haji Ahmed, Ayad Ahmad Mohammed

**Affiliations:** a Department of Internal Medicine, Duhok General Directorate of Health, Duhok City, Kurdistan Region, Iraq; b Department of Medicine, College of Medicine, University of Duhok, DUHOK, Kurdistan Region, Iraq; c Department of Internal Medicine, Non-communicable Disease Unit Duhok General Directorate of Health, Duhok City, Kurdistan Region, Iraq; d Department of Surgery, College of Medicine, University of Duhok, DUHOK, Kurdistan Region, Iraq

**Keywords:** Vitamin D, Type 2 diabetes, Glycemic control, Dyslipidemia, HbA1c

## Abstract

**Background:**

Vitamin D deficiency is prevalent in most parts of the world. Its insufficiency or deficiency is implicated in bone diseases, some cancers, infectious diseases, heart disease, autoimmune and metabolic diseases including type 2 diabetes mellitus.

**Results:**

The mean age of patients was 49.94 ± 9.36, while the mean age the controls was 48.95 ± 10.56. Females constituted 56.1% and males 43.9% in the cases group, while for the control group females were 54.8% and males were 43.9%. Low vitamin D levels were detected in 110 (71%) of cases and 63 (40.6%) of controls. There was a significant difference in vitamin D levels among cases and controls (p < 0.001), vitamin D level was lower among females compared to males, p < 0.001 and those living in urban areas compared to rural areas, p < 0.001, BMI and dyslipidemia had a significant effect on vitamin D levels among diabetics, p values 0.002 and < 0.001 respectively. The serum 25(OH)-D level was significantly lower in patients with poor glycemic control compared to those with good glycemic control and in patients with a diabetes duration greater than 5 years, p values < 0.001 and 0.002 respectively. No significant correlation was detected with age and smoking, p values 0.181 and 0.260 respectively.

**Conclusion:**

There is a high prevalence of hypo-vitaminosis D among patients with type-2 diabetes, particularly among patients with poor glycemic control and in those with longer diabetes durations. Vitamin-D deficiency is more prevalence in females, and those living in urban areas, those with obesity and patients with dyslipidemia.

## Highlights

• Vitamin D deficiency is prevalent in most parts of the world.
• There is a high prevalence of hypovitaminosis D among patients with type 2 diabetes.
• Vitamin D deficiency is implicated many diseases including type 2 diabetes mellitus.

## 1 Introduction

Diabetes is a group of metabolic diseases characterized by hyperglycemia resulting from defects in insulin secretion, insulin action, or both. Globally it is estimated that 422 million adults are living with diabetes mellitus, according to the latest 2016 data from the World Health Organization, this number is suspected to raise to 642 million by the end of 2040 [1,2].

The global prevalence of diabetes among adults over 18 years of age has risen from 4.7% in 1980 to 8.5% in 2014, type 2 diabetes mellitus constitutes 90% of the cases with no sex predominance [3].

Diabetes can be classified into the following general categories: Type 1 diabetes (due to β-cell destruction, usually leading to absolute insulin deficiency), type 2 diabetes (due to a progressive insulin secretory defect on the background of insulin resistance), gestational diabetes mellitus (GDM) (diabetes diagnosed in the second or third trimester of pregnancy that is not clearly overt diabetes), and specific types of diabetes due to other causes, e.g., monogenic diabetic syndromes (such as neonatal diabetes and maturity-onset diabetes of the young, diseases of the exocrine pancreas (such as cystic fibrosis), drug- or chemical-induced diabetes such as in the treatment of HIV/AIDS or after organ transplantation [2,4].

Vitamin D was originally classified as merely a vitamin but recently is classified as a hormone and involved in a plethora of physiological processes. Its insufficiency or outright deficiency is implicated in not only bone diseases but also numerous serious and often fatal diseases, including many cancers, infectious diseases, heart disease, autoimmune and metabolic diseases including type 2 Diabetes Mellitus (t2DM), vitamin D status has been verified to be inversely associated with future risks of t2DM [5].

Vitamin D deficiency is prevalent in most parts of the world, generally, humans receive vitamin D by being exposed to the sunlight or dietary intake, such as fish oil and nutritional supplements. There is an association between low vitamin D levels and decreased insulin sensitivity, vitamin D stimulates insulin production, low vitamin D concentrations are associated with a higher likelihood of the occurrence of diabetic complications, such as cardiovascular disease, renal impairment, and peripheral arterial disease [[6], [7], [8]].

A normal level of vitamin D is defined as a 25(OH)D concentration greater than 30 ng/mL (75 nmol/L), vitamin D insufficiency is defined as a 25(OH)D concentration of 20–30 ng/mL (50–75 nmol/L), vitamin D deficiency is defined as a 25(OH)D level less than 20 ng/mL (50 nmol/L) [9].

Vitamin D affects mechanisms related with metabolic syndrome and T2DM pathophysiology, including impaired B-cell function and insulin resistance, potentially by directly activating vitamin D receptors or by indirect effects by a calcium homeostasis regulation [10].

There is increasing evidence on association between vitamin D insufficiency and diabetes, but the impact of vitamin D status on glycaemic status and vice versa, has not been well reported.

The aim of this study is to study the levels of 25-hydroxyvitamin D (25(OH)D) and the relationship between 25(OH)D levels and glycemic control in patients with diabetes mellitus type 2.

## 2 Patients and methods

This is a case-control study conducted at the Diabetic Center of … … Teaching Hospital, … … … …, from the February 1, 2018 to the July 30, 2018. A total number of 310 participants were included, 155 with type 2 DM and 155 patients without type 2 DM from … … Governorate.

A questionnaire was administered to determine the demographics of the patients like age, sex and residency, diabetes duration, Smoking, body mass index (BMI) and dyslipidemia was examined. Selection of cases and healthy controls was carried out by using a random sampling technique. A verbal consent was obtained from all participants.BMI was calculated for each subject. None of the females was pregnant. Diabetes mellitus type 2 patients included in the study were on treatment either with diet only or with diet and oral antidiabetic drugs.

Initial consultation and examination involved the routine assessment of Serum 25(OH) D and glycaemic control (HbA1c) concentration, and also following laboratory parameters were done including (complete blood count, blood sugar, lipid profile, renal function test, liver function test). Serum concentration of 25(OH) D was measured by enzyme linked immunosorbent assay (ELISA) method. HbA1c was measured by spectrophotometer. Biochemical blood measurements were determined by a standard laboratory procedure using Cobas 6000, Roche/Hitachi. This was referred to the Central public health laboratory.

We used the following cut-off values for classifying vitamin D status as follow: A normal level of vitamin D is defined as a 25(OH)D concentration greater than 30 ng/mL (75 nmol/L), vitamin D insufficiency is defined as a 25(OH)D concentration of 20–30 ng/mL (50–75 nmol/L), and vitamin D deficiency is defined as a 25(OH)D level less than 20 ng/mL (50 nmol/L) [11].

Diabetes Mellitus was defined as documented by blood test, and/or use of anti-diabetic medications or new onset DM according to the American Diabetes Association (ADA) guidelines. Table 1.

**Table 1:** Criteria for the diagnosis of diabetes according to the latest American Diabetes Association (ADA) guidelines.

Diagnosis	A1C (Glycosylated hemoglobin) (percent)	Fasting plasma glucose (FPG) mgldl	2hr Oral glucose tolerance test (OGTT) mgldl	Random plasma glucose test (RPG) mg/dl
Normal	below 5.7	99 or below	139 or below	
Prediabetes	5.7 to 6.4	100 to 125	140 to 199	
Diabetes	6.5 or above	126 or above	200 or above	200 or above + symptoms of DM

Controls were matched to cases by age and sex and they should be free from DM. Controls were recruited from attendants or relative of non-diabetic patients from non-diabetic Center, or patients attending non diabetic outpatient clinics for disorders unrelated to DM.

A1C (Glycosylated hemoglobin) reﬂects average glycemia over several months and has strong predictive value for diabetes complications, furthermore, patients were classified according to their HbA1c level into the followings: <5.7% (normal), 5.7%–6.4% (prediabetes), > =6.5% (diabetes) and≥7% (uncontrolled Diabetes) [12].

Dyslipidemia was diagnosed based on AACE guidelines it referred to: Total cholesterol: desirable < 200 mg/dl, Borderline high 200–239, High > 239 mg/dl, high density lipoprotein –cholesterol: dyslipidemic Low < 40 mg/dl in males, < 50 mg/dl in females, low density lipoprotein -cholesterol: Optimal < 100 mg/dl, near optimal 100–129 mg/dL, Borderline high 130–159 mg/dl, High 160–189 mg/dl, very high > 189 mg/dl, triglyceride: Normal < 150 mg/d, High 150–199 mg/dl, and hypertriglyceridemic 200–499 mg/dl, very high > 499 mg/dl [13].

Body Mass Index (BMI): as a weight (Kg) divided by height (M^2^).BMI 18.5–24.9 were considered normal, while those with BMI 25–29.9 were considered overweight and those BMI ≥ 30 were considered obese [14].

**Inclusion criteria for cases were:** Age more than 25 years old and Type 2DM on treatment either with diet only or with diet and oral antidiabetic drugs.

**Exclusion criteria of cases were:** Patients with end stage renal disease (ESRD), patients with chronic liver disease, patients with malignancies, patients with bone diseases, patients with any history of the use of drugs such as insulin, anticonvulsants, vitamin D or calcium supplements, patients with anaemia (Iron deficiency, Hemoglobinopathies, Hemolytic etc), patients with Type 1 diabetes and pregnancy.

## 3 Statistical analyses

The Statistical Package for Social Sciences (SPSS version 21) was used for data analysis, and data were presented as mean ± standard deviation. The descriptive statistics of participants were obtained by determining frequency distributions of categorical data and weighted means and standard errors of continuous variables, such as age, gender, BMI, dyslipidemia, DM, smoking and serum vitamin D. Significant differences in categorical and continuous variables between the cases and control groups were analyzed using the chi-squared test. Level of statistical significance was set at < 0.05.

The research is registered according the World Medical Association's Declaration of Helsinki 2013 at the research registry at the 19th of September 2020, Research registry UIN: research registry **6495**.

**The work of this article has been reported in line with the STROCSS criteria.** [15].

## 4 Results

The patents demographic characteristics and the results of the vitamin D level, diabetes and BMI are shown in Table 2.

**Table 2:** Baseline characteristics of participants.

Categories	Cases (n = 155) Frequency and percentage	Controls (n = 155) Frequency and percentage
Age in years (M; SD)	49.94 ± 9.361	48.95 ± 10.525
Sex Male Female	68 (43.9) 87 (56.1)	70 (45.2) 85 (54.8)
Age <40 years >40 years	22 (14.2) 133 (85.8)	40 (25.8) 115 (74.2)
Residency Rural Urban	44 (28.4) 111 (71.6)	59 (38.1) 96 (61.9)
Smoking Non smoker Ex-smoker Current smoker	123 (79.4) 3 (1.9) 29 (18.7)	144 (92.9) 2 (1.3) 9 (5.8)
BMI <18.5 18.5–24.9 25–29.9 30–39.9	0 (0) 48 (31) 83 (53.5) 24 (15.5)	1 (0.6) 54 (34.8) 95 (61.3) 5 (3.2)
Dyslipidemia Yes Non	67 (43.2) 88 (56.8)	8 (5.2) 147 (94.8)
FBS <100 100–125 ≥126	0 (0) 20 (12.9) 135 (87.1)	118 (76.1) 37 (23.9) 0 (0)
HbA1C <5.7% 5.7%–6.4% ≥ 6.5% >7%	0 (0) 0 (0) 43 (27.7) 112 (72.3)	126 (81.3) 29 (18.7) 0 (0) 0 (0)
Vitamin D level Deficient Insufficient Normal	50 (32.3) 60 (38.7) 45 (29)	23 (14.8) 40 (25.8) 92 (59.4)

**Fig. 1:**
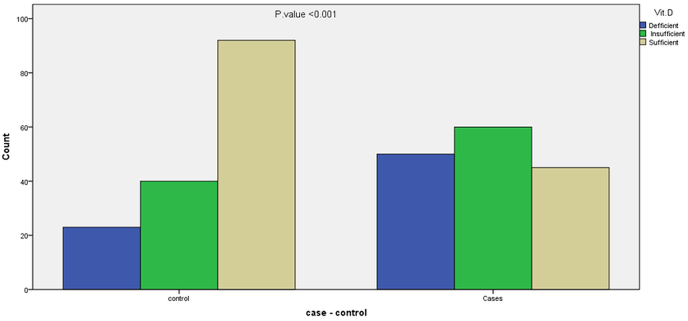
Distribution of vitamin D Status among cases and controls.

During the enrolment period of six months, 155 patients with DM type2 from Diabetic Center and 155 controls were included in this study. The patients mean age was 49.94 ± 9.361SD, while the controls mean age was 48.95 ± 10.525 SD. Females were 87 (56.1%) and males were 68 (43.9%) in cases, while females were 85 (54.8%) and males were 70 (43.9) in controls. Vitamin D insufficient and deficient were 110 (71%) in cases and 63 (40.6%) in controls as shown in Table 3 and Fig. 1.

**Table 3:** Relation of characteristics of cases with vitamin D status.

Variable	Vitamin D level			Total	P value
	Deficiency (n = 50)	Insufficiency (n = 60)	Sufficiency (n = 45)		
Sex Male Female	9 (18.0) 41 (82.0)	28 (46.7) 32 (53.3)	31 (68.9) 14 (31.1)	68 (43.9) 87 (56.1)	**<0.001**
Age <40 ≥40	5 (10.0) 45 (90.0)	7 (11.7) 53 (88.3)	10 (22.2) 35 (77.8)	22 (14.2) 133 (85.8)	0.181
Residency Rural Urban	5 (10.0) 45 (90.0)	9 (15.0) 51 (85.0)	30 (66.7) 15 (33.3)	44 (28.4) 111 (71.6)	**<0.001**
Duration of DM <5 years >5 years	15 (30.0) 35 (70.0)	24 (40.0) 36 (60.0)	29 (64.4) 16 (35.6)	68 (43.9) 87 (56.1)	**0.002**
Smoking Non smoker Ex-smoker Current smoker	44 (88.0) 1 (2.0) 5 (10.0)	45 (75.0) 2 (3.3) 13 (21.7)	34 (75.6) 0 (0.0) 11 (24.4)	123 (79.4) 3 (1.9) 29 (18.7)	0.260
BMI 18.5–24.9 25–29.9 30–39.9	7 (14.0) 25 (50.0) 18 (36.0)	18 (30.0) 36 (60.0) 6 (10.0)	23 (51.1) 22 (48.9) 0 (0.0)	48 (31.0) 83 (53.5) 24 (15.5)	**<0.001**
FBS 100–125 ≥126	3 (6.0) 47 (94.0)	7 (11.7) 53 (88.3)	10 (22.2) 35 (77.8)	20 (12.9) 135 (87.1)	0.058
HbA1C ≥ 6.5% >7%	6 (12.0) 44 (88.0)	12 (20.0) 48 (80.0)	25 (55.6) 20 (44.4)	43 (27.7) 112 (72.3)	**<0.001**
Dyslipidemia Yes Non	35 (70.0) 15 (30.0)	29 (48.3) 31 (51.7)	3 (6.7) 42 (93.3)	67 (43.2) 88 (56.8)	**<0.001**

There was no significant difference in the age and sex between two groups as age and sex were matched. The patients had a higher prevalence of obesity and smoking status, and there was significant difference between two groups (p = 0.002).There was highly significant difference in distribution of dyslipidemia and vitamin D status among cases and controls (p < 0.001) as shown in Table 3.

Relation of characteristics of cases with vitamin D status has been shown in (Table 3).Among the different age groups, smoking status, the prevalence of low vitamin D levels was not significant as compared to that of sufficient levels.(p = 0181,0260) respectively. While regarding to FBS slightly significant (p = 0.058).

Vitamin D level was low (insufficient and deficient) in Female as compared to male, the statistical analysis showed highly significant effect (p < 0.001). Distribution of study groups according to residency, in our study vitamin D is significantly low in those living in Urban as compared to Rural (p < 0.001). Similarly, BMI and dyslipidemia had highly significant effect (p < 0.001) on vitamin D level among diabetes patients.

The serum 25(OH) D level was significantly lower (p < 0.001) for patients with poor glycaemic control as compared to that for good glycaemic control diabetics. A significant difference was also noticed between values for serum 25(OH) D level of patients with a diabetes duration of more than 5 years and of those with a duration less than 5 years (p = 0.002).

## 5 Discussion

This study show that there was a highly significant difference in vitamin D concentrations between type2DM and healthy controls. Our data suggest that the vitamin D insufficiency and deficiency was prevalent in both cases (71%) and controls (40.6%), however we found a significant different (p < 0.001), such a high prevalence of low vitamin D status is worth mentioning. It is favorably comparable to values from USA by Paul A. Oakley et al. they found 74% hypovitminD in type2DM as compared to control. **A case control study was done by** Moradzadeh K et al. in Iran, they found significant difference between type2DM and healthy control. Paulo et al. found high prevalence (62%) of hypovitaminosis D among Brazilians with type 2 diabetes as compared to control. However, Nielsen, et al. from United States did not support an association between low vitamin D levels and risk of type 2 diabetes [4,[16], [17], [18], [19]].

Several hypotheses have tried to explain the relation between Vitamin D deficiency and type2DM. Abdul-Ghani et al. showed **in a population based study** that Vitamin D play an important role in insulin resistance as well as impaired beta cell function, the main issues with t2DM. Once theorized that glucose tolerance could adversely affect insulin sensitivity and beta-cell function, this was directly proven by Chiu et al. in 126 glucose-tolerant subjects in their studies [6,20,21].

Our sample did not demonstrate any significant age and vitamin D level association. Age, however would be expected to negatively correlate with vitamin D status since older individuals are less efficient at producing vitamin D in sunlight as opposed to younger individuals as well as the fact that older individuals kidneys are less able to convert vitamin D into its active form [22].

25-Hydroxyvitamin D levels are lower in smoking persons when compared with non-smoker persons. The explanation may be due to Smoking decreases Calcium and injures the body. Our study showed lower serum 25-hydroxyvitamin D levels in smoking patients but this difference was not significant, which may be due to the small sample of study [22].

25-Hydroxyvitamin D levels are lower in obese persons when compared with lean persons. The explanation may be due to lower levels of exercise and sunlight exposure and sequestering of 25-hydroxyvitamin D in adipose tissues. Our study showed lower serum 25-hydroxyvitamin D levels in obese patients and this difference was highly significant [[23], [24], [25]].

Dyslipidemia was an independent predictor of hypovitaminosis D and total cholesterol was inversely correlated with levels of 25(OH) D. we found that dyslipidemia had highly significant effect (p < 0.001) on vitamin D level among diabetes patients. Our data are consistent with another study in the literature that evaluated metabolic parameters in type 2 diabetes patients from sunny regions [26].

Our data suggest that low vitamin D (insufficient and deficient) was significantly more in females’ patients; this might be due to social behaviors and due to religious reasons, the women must cover all the body with cloths, and wear a hijab when they go outdoors. Clothes are a main blocker to sun exposure and therefore 25(OH) D synthesis and status. Sun exposure to uncovered face and hands as in hijab dressed females is not enough for vitamin D synthesis, even when they live in a sunny climate. This finding was in accordance with the results that reported by many other investigators [27,28].

Distribution of study groups according to residency, in our study vitamin D was significantly low in those living in Urban as compared to Rural (p < 0.001).The reason behind those results might be the different lifestyles and physical activity [27].

The serum 25(OH) D level was significantly lower in patients with poor glycemic control as compared to that in good glycemic control diabetics. A significant difference was also noticed between values for serum 25(OH) D level of patients with a diabetes duration of more than 5 years and of those with a duration less than 5 years. In the present study, an inverse association between 25(OH) D and HbA1c in diabetes mellitus type 2 patients noted. This observation reflects the additive effect of glycemic control on vitamin D status. Although the significance of vitamin D deficiency in type2 DM is well established in many observational studies, the role of vitamin D supplementation on diabetes is not clear. A recent meta-analysis that reported that there is insufficient evidence of a beneficial effect to recommend vitamin D supplementation as a means of improving glycemic control in patients with type 2 diabetes. Most studies that investigated the effect of vitamin D supplementation in patients with type 2 diabetes did not find a significant effect on glycemic control. However, **in one case control study that was performed by** Nikooyeh et al. in which patients were randomized into plain yogurt drink or vitamin D3–fortified yogurt drink (~1000 IU/day), after 12 weeks, there was a significant reduction in HbA_1c_, fasting insulin, and glucose. Results from randomized trials on the effect of vitamin D and/or calcium supplementation on insulin resistance show improvement of insulin action with supplementation. A study from Iran showed that the effect of vitamin D was to reduce FPG 30% after patients received 50,000 unit of vitamin D3 orally per week for eight weeks [[29], [30], [31], [32], [33], [34]].

Despite the scientific merit, this study has some limitations, including small sample size of the patients and controls, the referral biases of single center experience and difficult to follow up patients.

## 6 Conclusions

In conclusion, we have identified a high prevalence (71%) of hypovitaminosis D among patients with type 2 diabetes, particularly among patients with poor glycemic control and in those with longer diabetes durations. Also we found that vitamin D deficiency was more prevalence in females gender, those living in urban, obesity and dyslipidemia **Recommendations:** Routine screening of vitamin D status in all patients with DM type2 and attention should be paid to general population as vitamin D deficiency was prevalence in more than half of our population. Further larger studies are recommended to overcome the study limitations including small sample size.

## Declaration of interest statement

There is no any conflict of interest to be declared.

## References

[1] Organization W.H. (2016). Global Report on Diabetes.

[2] Association A.D. (2010). Diagnosis and classification of diabetes mellitus. Diabetes Care.

[3] Organization W.H., WH (2016). Global Report on Diabetes.

[4] Najeeb H.A. (2020). Parental history of coronary artery disease among adults with hypothyroidism: case controlled study. Annals of Medicine and Surgery.

[5] Khan H. (2013). Vitamin D, type 2 diabetes and other metabolic outcomes: a systematic review and meta-analysis of prospective studies. Proc. Nutr. Soc..

[6] Chiu K.C. (2004). Hypovitaminosis D is associated with insulin resistance and β cell dysfunction. Am. J. Clin. Nutr..

[7] Gaddipati V.C. (2011). The relationship of vitamin D status to cardiovascular risk factors and amputation risk in veterans with peripheral arterial disease. J. Am. Med. Dir. Assoc..

[8] Dogan Y. (2015). 25-Hydroxy-vitamin D level may predict presence of coronary collaterals in patients with chronic coronary total occlusion. Postępy w Kardiologii Interwencyjnej= Advances in Interventional Cardiology.

[9] Holick M.F. (2007). Vitamin D deficiency. N. Engl. J. Med..

[10] Pittas A.G. (2007). The role of vitamin D and calcium in type 2 diabetes. A systematic review and meta-analysis. J. Clin. Endocrinol. Metab..

[11] Holick M.F. (2011). Evaluation, treatment, and prevention of vitamin D deficiency: an Endocrine Society clinical practice guideline. J. Clin. Endocrinol. Metab..

[12] Association A.D. (2017). 2. Classification and diagnosis of diabetes. Diabetes Care.

[13] Jellinger P. (2012). American Association of Clinical Endocrinologists' guidelines for management of dyslipidemia and prevention of atherosclerosis. Endocr. Pract..

[14] Eckert S., Kohler S. (2014). Urbanization and health in developing countries: a systematic review. World Health Popul..

[15] Agha R. (2019). STROCSS 2019 Guideline: strengthening the reporting of cohort studies in surgery. Int. J. Surg..

[16] Moradzadeh K. (2008). Normative values of vitamin D among Iranian population: a population based study. International Journal of Osteoporosis & Metabolic Disorders.

[17] Rolim M.C. (2016). Relationship between vitamin D status, glycemic control and cardiovascular risk factors in Brazilians with type 2 diabetes mellitus. Diabetol. Metab. Syndrome.

[18] Nielsen N.O. (2016). Associations between Vitamin D status and type 2 diabetes measures among Inuit in Greenland may be affected by other factors. PloS One.

[19] Qasim B.A., Mohammed A.A., Ahmed M.J. (2019). Lipid profile IN subclinical hypothyroidism: a two centers experience. Duhok Medical Journal.

[20] Abdul-Ghani M.A., Tripathy D., DeFronzo R.A. (2006). Contributions of β-cell dysfunction and insulin resistance to the pathogenesis of impaired glucose tolerance and impaired fasting glucose. Diabetes Care.

[21] Rossetti L., Giaccari A., DeFronzo R.A. (1990). Glucose toxicity. Diabetes Care.

[22] Health N.I.o. (2011). Dietary Supplement Fact Sheet: Vitamin D. http://www.%20dietary-supplements.%20info.%20nih.%20gov/factsheets/vitamind.%20asp.

[23] Rosen C.J. (2011). Vitamin D insufficiency. N. Engl. J. Med..

[24] Ahmed S.S., Mohammed A.A. (2020). Effects of thyroid dysfunction on hematological parameters: case controlled study. Annals of Medicine and Surgery.

[25] Qasim B. (2018). Dyslipidemia in subclinical hypothyroidism: a case-control study. J Endocrinol Diab.

[26] Yu J.R. (2012). Serum vitamin d status and its relationship to metabolic parameters in patients with type 2 diabetes mellitus. Chonnam medical journal.

[27] Miettinen M.E. (2014). Association of serum 25-hydroxyvitamin D with lifestyle factors and metabolic and cardiovascular disease markers: population-based cross-sectional study (FIN-D2D). PloS One.

[28] Najeeb H.A. (2020). Vitamin D level and endogenous DNA damage in patients with cancers in Duhok city, KRG-Iraq. Annals of Medicine and Surgery.

[29] George P., Pearson E., Witham M. (2012). Effect of vitamin D supplementation on glycaemic control and insulin resistance: a systematic review and meta‐analysis. Diabet. Med..

[30] Heshmat R. (2012). Effect of vitamin D on insulin resistance and anthropometric parameters in Type 2 diabetes; a randomized double-blind clinical trial. Daru.

[31] Jorde R., Figenschau Y. (2009). Supplementation with cholecalciferol does not improve glycaemic control in diabetic subjects with normal serum 25-hydroxyvitamin D levels. Eur. J. Nutr..

[32] Soric M., Renner E., Smith S. (2012). Effect of daily vitamin D supplementation on HbA1c in patients with uncontrolled type 2 diabetes mellitus. J. Diabetes.

[33] Nikooyeh B. (2011). Daily consumption of vitamin D–or vitamin D+ calcium–fortified yogurt drink improved glycemic control in patients with type 2 diabetes: a randomized clinical trial. Am. J. Clin. Nutr..

[34] Pittas A.G. (2007). The effects of calcium and vitamin D supplementation on blood glucose and markers of inflammation in nondiabetic adults. Diabetes Care.

